# Team-, case-, lecture- and evidence-based learning in medical postgraduates training

**DOI:** 10.1186/s12909-024-05650-5

**Published:** 2024-06-18

**Authors:** Tianlong Huang, Shun Zhou, Qiaoyan Wei, Chun Ding

**Affiliations:** Department of Ophthalmology, The Second Xiangya Hospital of Central South University, 139 Renmin Middle Road, Changsha, 410011 China

**Keywords:** Team-, case-, lecture-, evidence-based learning, Lecture-based learning, Medical postgraduates, Literature review writing, Theoretical examination

## Abstract

**Background:**

The aim of this study was to evaluate the effectiveness of team-, case-, lecture-, and evidence-based learning (TCLEBL) methods in cultivating students’ clinical and research abilities, as compared to traditional lecture-based learning (LBL) approaches.

**Methods:**

Forty-one medical postgraduates were divided into two groups, a TCLEBL group and an LBL group. Teaching effectiveness was evaluated through student- and teacher-feedback questionnaires, scores from theoretical examinations and written literature reviews, and student learning burdens.

**Results:**

Compared to the LBL approach, both teachers and students were more satisfied with the TCLEBL model (*p* < 0.001 for both teachers and students). The TCLEBL group performed significantly higher on the theory test compared to the LBL group (*p* = 0.009). There were significant differences between the LBL and TCLEBL groups, respectively, in terms of literature review and citations (12.683 ± 2.207 vs. 16.302 ± 1.095, *p* < 0.001), argument and perspective (12.55 ± 1.572 vs. 16.333 ± 1.354, *p* < 0.001), comprehensiveness of content (13.3 ± 2.268 vs. 16.683 ± 1.344, *p* < 0.001), and scientific rigor and accuracy (10.317 ± 1.167 vs. 12.746 ± 0.706, *p* < 0.001). There was no significant difference in the total extracurricular time expended between the two groups (323.75 ± 30.987 min vs. 322.619 ± 24.679 min, respectively for LBL vs. TCLEBL groups, *p* = 0.898).

**Conclusions:**

TCLEBL is an effective teaching method that cultivates students’ clinical and research abilities.

## Background

The quality of medical postgraduate training plays a defining role in shaping the future of healthcare [1]. Traditional, lecture-focused models emphasize the theoretical aspects of knowledge acquisition but fail to cultivate self-directed learning in trainees [2]. Meanwhile, clinical instruction stresses skill mastery but rarely explores complex cases, potentially limiting development of trainees’ critical thinking and problem-solving abilities [3].

As the next generation of healthcare professionals, postgraduates should possess certain research competencies. It is crucial to prioritize the cultivation of an inquisitive mindset. This can be achieved through engaging activities such as formulating research questions, creating study protocols, and staying updated with the latest evidence by critically evaluating high-quality clinical trials. Trainees must also acquire essential research methodologies and skills, including literature searching and critical appraisal. These abilities will enable them to promptly address any challenges they may encounter in their future practice [4–9]. Educational systems that are adaptable and diverse have the potential to unlock the full capabilities of postgraduates in advancing healthcare innovation and delivery. Merely relying on didactic lectures is inadequate for fostering self-directed learning and cultivating well-rounded, expert clinicians and scientists in the future. It is important to incorporate interactive and experiential learning approaches that encourage critical thinking, problem-solving, and creativity. By embracing a variety of teaching methods and providing opportunities for hands-on experiences, postgraduates can truly thrive and contribute to the advancement of healthcare.

Team-based learning (TBL) is gaining popularity in medical postgraduate education. TBL emphasizes collaborative learning within groups rather than individual study. By promoting team spirit and problem-solving skills through coordinated thinking, TBL can effectively enhance the quality and efficiency of learning compared to traditional didactic approaches. TBL places greater emphasis on the learning process itself rather than solely focusing on outcomes. This approach allows for active engagement, critical thinking, and effective communication among team members, leading to a deeper understanding of the subject matter. Ultimately, TBL has the potential to contribute to the overall improvement of learning outcomes in medical postgraduate education [10, 11].

Case-based learning (CBL) is an effective approach that enhances clinical analytics and problem-solving skills by using authentic or hypothetical cases to stimulate self-driven learning. This method encourages active engagement and critical thinking rather than passive study, aiding in the internalization of skills. Educators play a crucial role in this process by pre-selecting representative clinical vignettes that guide trainees in understanding key diagnoses and making informed judgments. Small groups of students then delve deeper into the cases through multi-perspective and self-reflective thinking, facilitated by the teacher. This approach allows for a comprehensive exploration of the cases and ensures a well-rounded learning experience for the trainees [12–16].

Evidence-based learning is a major focus in current medical education reform. This approach emphasizes using reliable evidence as the foundation for study, which is crucial for enhancing the research capabilities of postgraduates. In the past, certain content in medical education may have been influenced more by subjective opinions. However, evidence-based learning models aim to ensure that judgments and decisions are grounded in sound evidence, aligning closely with clinical practice and research pursuits. By incorporating evidence-based learning into postgraduate education, trainees are encouraged to critically evaluate research findings, stay updated on the latest evidence, and apply evidence-based approaches in their future practice. This helps to cultivate a strong research caliber and ensures that postgraduates are well-equipped to contribute to the advancement of medical knowledge and patient care [17–19].

While traditional didactic instruction may have certain limitations, completely abolishing didactics would be misguided. Didactic instruction plays a vital role in providing a structured and systematic delivery of medical knowledge frameworks. Through lectures, instructors can analyze prototypical cases and present principles that help trainees quickly grasp important concepts and their practical applications. This form of instruction serves as an effective conduit for disseminating foundational knowledge and providing a solid framework for further learning and clinical practice. It should be regarded as a complementary approach alongside other interactive and experiential learning methods, rather than being disregarded entirely [20].

The team-, case-, lecture-, and evidence-based learning (TCLEBL) instructional method, by integrating the strengths of various approaches, aims to provide a comprehensive, well-rounded teaching experience in medical postgraduate education [21]. However, there is currently a lack of research and reports on the application of this amalgamated methodology specifically in medical postgraduate education. It is important to unify different pedagogies to optimize use of their respective merits and maximize learning outcomes. However, the implementation and impact of this approach need to be documented and analyzed. Therefore, the objective of this study is to evaluate the effectiveness of the TCLEBL method in cultivating students’ clinical and research abilities, in comparison to traditional lecture-centered teaching approaches.

## Methods

### Research subjects

This research adhered to the Helsinki Declaration and was approved by the Ethics Committee of the Second Xiangya Hospital. A total of 41 postgraduate students in ophthalmology were enrolled and divided into the TCLEBL group (*n* = 21) and the traditional lecture-based learning (LBL) group (*n* = 20). Postgraduate entrance examination scores were used to assess students’ fundamental learning abilities (*p* = 0.497).

### Study design

Ocular toxocariasis (OT) was chosen as the case study for this research. OT is an infectious parasitic disease that primarily affects children and has a certain incidence rate. Although OT is not included in the content covered in standard five-year ophthalmology textbooks, it is a disease in which ophthalmology graduate students must be proficient. OT presents with distinct clinical manifestations and poses diagnostic and treatment challenges. Therefore, it serves as an ideal case to evaluate the effectiveness of the TCLEBL method in conveying knowledge about this particular condition to medical students.

In the LBL group, we adopted a traditional didactic teaching approach. Relevant materials were distributed to students before the class. During the class, the instructor explained the topic by integrating clinical cases, providing a systematic explanation of various aspects of OT, including its definition, clinical manifestations, auxiliary examinations, diagnosis, and treatment. Then, the students were guided to retrieve relevant literature from academic databases, medical journals, and specialty organization guidelines. After the class, the students were required to complete a review article on OT.

**Fig. 1:**
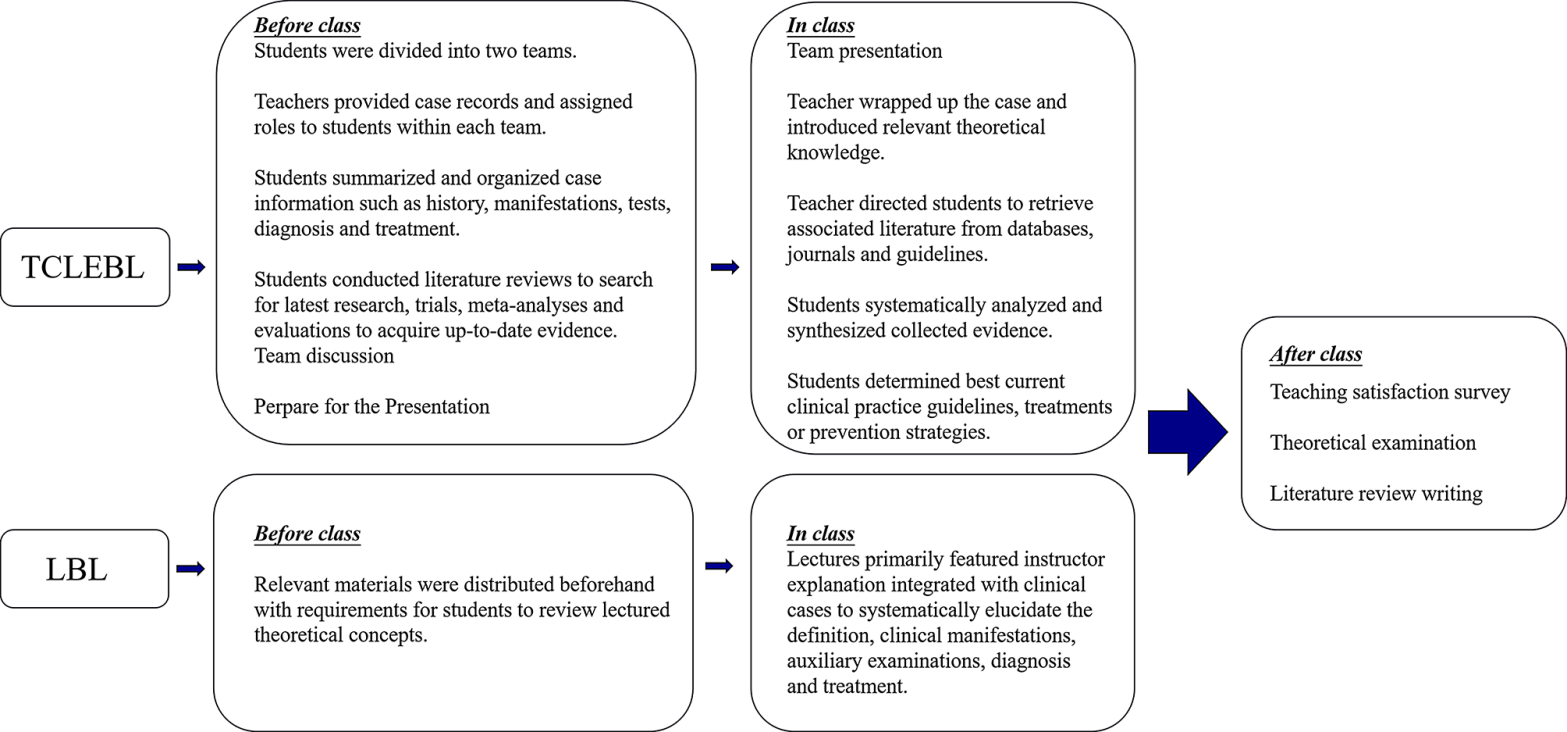
Flowcharts of TCLEBL and LBL teaching models. TCLEBL: team-, case-, lecture-, evidence-based learning; LBL: lecture-based learning

For the TCLEBL group, students were divided into two teams. Before class, teachers provided complete case records and assigned individual roles within teams to summarize and organize history, manifestations, auxiliary tests, diagnosis, and treatment; while conducting extensive literature reviews to search for the latest scientific research, clinical trials, meta-analyses, and systematic evaluations to acquire up-to-date evidence and results. During class, both teams presented diagnostic and treatment plans for the case, applying theoretical and evidentiary resources and engaging in discussions for comprehensive exchange of perspectives with teacher guidance. The teacher wrapped-up the case while fully introducing the relevant theoretical knowledge framework, then directed students to retrieve associated literature from academic databases, medical journals, and specialty organization guidelines. Collected evidence underwent systematic analysis and synthesis to determine current best clinical practice guidelines, treatments, or preventive strategies. After the class, the students were also required to complete a review article on OT. Details were shown in the Fig. 1.

### Evaluation

The same teacher instructed both groups with consistent teaching content.

Teaching satisfaction assessment: Anonymous questionnaires were distributed to teachers and all students after course completion to self-evaluate teaching quality using a 10-point visual analogue scale (VAS), with 0 indicating “no effect” and 10 indicating “completely perfect.” For students, items included: classroom interactivity, learning efficiency, level of knowledge mastery, self-directed learning ability, teamwork skills, preferences for this teaching mode, extracurricular time commitment, improved clinical reasoning abilities, and improved research abilities. For teachers, items included: “The lecture greatly enhances students’ understanding about this topic,” “The class met my expectations,” “It is an enjoyable way of teaching,” “Overall, I am satisfied with the quality of this class,” and “The climate of this class is conducive to learning for students.”

Theory examination: To precisely reflect long-term learning outcomes and in-depth understanding of OT, a theoretical examination was administered after trainees had undergone six weeks of a clinical practicum. A theoretical exam assessing concepts, clinical manifestations, auxiliary examinations, diagnosis, and treatment of OT was designed, incorporating multiple choice, judgment, and open-response questions, scored out of a total 100 points.

Literature-review writing was assessed in six areas: structure and organization, literature review and citations, argument and perspective, comprehensiveness of content, scientific rigor and accuracy, and clarity of expression and language fluency, for a total score of 100 points. Blind evaluation was conducted by three instructors, and the average score was taken as the final score.

The two groups of graduate students needed to report their pre-class preparation time, post-class review time, and time spent writing literature reviews.

### Statistical analysis

Statistical analyses were performed using SPSS 26.0 software. The measurement data are expressed as the mean ± standard deviation. Demographic data of the residents were analyzed using an independent *t*-test or χ^2^ test. Students’ questionnaire data were analyzed using an independent *t*-test. Teachers’ questionnaire data were analyzed using an paired *t*-test. The theoretical exam scores, the hours spent on class preparation, review, and writing the literature review were compared between the two groups by an independent *t*-test. A *p* value of less than 0.05 was considered statistically significant.

## Result

A total of 41 postgraduate students (males = 15, females = 26) were recruited for this study. The LBL group consisted of 20 students (males = 8, females = 12); the TCLEBL group included 21 students (males = 7, females = 14). As shown in Table 1, the two groups of medical postgraduates did not exhibit any statistically significant differences in terms of age (23.2 ± 0.894 vs. 23.238 ± 0.768, *p* = 0.884), gender distribution (*p* = 0.658), or scores on the postgraduate entrance examination (394.5 ± 7.287 vs. 392.381 ± 11.843, *p* = 0.497).

**Table 1:** Demographic information of medical postgraduates

	LBL	TCLEBL	t/χ^2^	*P*
Number of students	20	21	-	-
Gender			0.196	0.658
Male	8	7		
Female	12	14		
Age (years), mean ± SD	23.2 ± 0.894	23.238 ± 0.768	0.147	0.884
Postgraduate Entrance Examination Scores	394.5 ± 7.287	392.381 ± 11.843	-0.686	0.497

TCLEBL: team-, case-, lecture-, evidence-based learning; LBL: lecture-based learning

The results of the student survey are summarized in Table 2. Statistical analysis showed that students in the TCLEBL group rated significantly higher than the LBL group in terms of classroom interactivity (7.714 ± 0.902 vs. 5.75 ± 0.91, *p* < 0.001), learning efficiency (6.857 ± 1.062 vs. 4.1 ± 0.912, *p* < 0.001), knowledge mastery (7.571 ± 0.746 vs. 4.5 ± 0.889, *p* < 0.001), self-directed learning ability (7.476 ± 0.928 vs. 5.3 ± 0.923, *p* < 0.001), teamwork skills (7.667 ± 0.856 vs. 5.45 ± 0.999, *p* < 0.001), students’ preference for this teaching model (7.762 ± 0.889 vs. 4.75 ± 0.851, *p* < 0.001), improved clinical reasoning abilities (6.238 ± 0.625 vs. 4.1 ± 0.968, *p* < 0.001), and improved research capacities (8.048 ± 0.669 vs. 5.45 ± 0.826, *p* < 0.001).

**Table 2:** The questionnaire survey was collected from the two groups of medical postgraduates

Items	LBL	TCLEBL	t	*P*
Classroom interactivity	5.75 ± 0.91	7.714 ± 0.902	6.937	< 0.001
Learning efficiency	4.1 ± 0.912	6.857 ± 1.062	8.897	< 0.001
Level of knowledge mastery	4.5 ± 0.889	7.571 ± 0.746	12.007	< 0.001
Self-directed learning ability	5.3 ± 0.923	7.476 ± 0.928	7.522	< 0.001
Teamwork skills Students’ preference for this teaching mode Extracurricular time commitment	5.45 ± 0.999 4.75 ± 0.851 7.05 ± 0.605	7.667 ± 0.856 7.762 ± 0.889 6.762 ± 0.768	7.642 11.073 -1.330	< 0.001 < 0.001 0.191
Improved clinical reasoning abilities	4.1 ± 0.968	6.238 ± 0.625	8.445	< 0.001
Improved research abilities	5.45 ± 0.826	8.048 ± 0.669	11.094	< 0.001

TCLEBL: team-, case-, lecture-, evidence-based learning; LBL: lecture-based learning

Table 3 summarizes the teachers’ feedback. Compared to the LBL group, teachers felt that the TCLEBL model was more effective in enhancing students’ understanding of the topic (7.6 ± 0.699 vs. 6.1 ± 0.568, *p* < 0.001) and that the class met their expectations (8.2 ± 0.789 vs. 5.3 ± 0.483, *p* < 0.001). Furthermore, the teachers preferred the class climate in the TCLEBL class (7.8 ± 0.632 vs. 4.5 ± 0.527, *p* < 0.001) and were satisfied with the quality of this class (7.3 ± 0.675 vs. 5.4 ± 0.516, *p* < 0.001). Additionally, the teachers enjoyed the TCLEBL teaching method (7.9 ± 0.738 vs. 5.3 ± 0.675, *p* < 0.001).

**Fig. 2:**
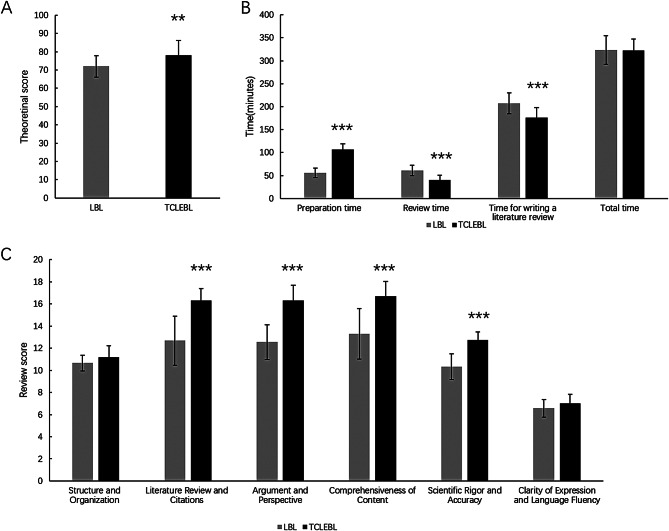
Comparison of medical postgraduates’ feedback between the TCLEBL group and the LBL group. (**A**) The TCLEBL group performed significantly higher on the theory test compared to the LBL group. (**B**) There was no significant difference in the total extracurricular time expended between the two groups. (**C**) The literature review was compared based on each scoring criterion. *p < 0.05, **p < 0.01, ***p < 0.001 compared with LBL. TCLEBL: team-, case-, lecture-, evidence-based learning; LBL: lecture-based learning

Results of the theoretical examination in Fig. 2A demonstrated that the average score of the TCLEBL group was 78.095 ± 8.148 points, while the average score of the LBL group was 71.951 ± 5.844 points. The TCLEBL group performed significantly higher on the theory test compared to the LBL group (*p* = 0.009).

**Table 3:** The questionnaire survey was collected from the two groups of medical postgraduates

Iems	LBL	TCLEBL	t	*P*
The lecture greatly enhances students’ understanding about this topic. The class met my expectations. It is an enjoyable way of teaching.	6.1 ± 0.568 5.3 ± 0.483 4.5 ± 0.527	7.6 ± 0.699 8.2 ± 0.789 7.8 ± 0.632	4.881 16.155 15.461	< 0.001 < 0.001 < 0.001
Overall, I am satisfied with the quality of this class.	5.4 ± 0.516	7.3 ± 0.675	6.862	< 0.001
The climate of this class is conducive to learning for students.	5.3 ± 0.675	7.9 ± 0.738	7.005	< 0.001

TCLEBL: team-, case-, lecture-, evidence-based learning; LBL: lecture-based learning

As shown in Fig. 2C, the literature review was compared between the groups based on each scoring criterion. There were significant differences between the LBL and TCLEBL groups, respectively, in terms of literature review and citations (12.683 ± 2.207 vs. 16.302 ± 1.095, *p* < 0.001), argument and perspective (12.55 ± 1.572 vs. 16.333 ± 1.354, *p* < 0.001), comprehensiveness of content (13.3 ± 2.268 vs. 16.683 ± 1.344, *p* < 0.001), and scientific rigor and accuracy (10.317 ± 1.167 vs. 12.746 ± 0.706, *p* < 0.001). However, there were no statistically significant differences in the scores for structure and organization (10.667 ± 0.717 vs. 11.175 ± 1.047, *p* = 0.079) and clarity of expression and language fluency (6.567 ± 0.81 vs. 7.016 ± 0.806, *p* = 0.083).

Figure 2B illustrates that the TCLEBL group had a significant increase in pre-class preparation time compared to the LBL group (55.75 ± 10.166 vs. 106.667 ± 12.383, *p* < 0.001). However, the TCLEBL group spent significantly less time on post-class review (60.5 ± 11.459 vs. 40.238 ± 10.305, *p* < 0.001) and writing the literature review (207.5 ± 22.682 vs. 175.714 ± 22.265, *p* < 0.001) compared to the LBL group. As a result, there was no significant difference in the total extracurricular time expended between the two groups (323.75 ± 30.987 vs. 322.619 ± 24.679, *p* = 0.898).

## Discussion

This study pioneered applying the TCLEBL model to ophthalmology postgraduate education. Teaching effectiveness was evaluated through questionnaires administered to teachers and students, post-course theory examinations, and assessments of literature review papers.

According to a post-course survey of students, The TCLEBL teaching method scored significantly higher than traditional teaching in terms of classroom interactivity, learning efficiency, knowledge acquisition, independent learning ability, teamwork skills, student preference for the model, and development of clinical thinking and research capacities. Firstly, TCLEBL enhances classroom interactivity, promoting active communication and collaboration among students. This interaction contributes to improving learning efficiency as students become more engaged and gain a deeper understanding of the learning content. Secondly, TCLEBL helps students better grasp knowledge. Through pre-class independent assignments and group discussions, students strengthen their understanding and application of knowledge through collaboration. This learning approach cultivates student independent learning abilities, enabling them to actively explore and expand their knowledge domains. Additionally, TCLEBL significantly improves student teamwork and collaboration skills. Group collaboration is at the core of TCLEBL, where students learn coordination, expression of viewpoints, and respect for others by jointly solving problems and discussing cases with their peers. This is a key advantage of the TBL teaching model [10, 22, 23]. Students’ preference for TCLEBL is also strengthened. In comparison to passive traditional learning, TCLEBL emphasizes student participation and active learning, making the learning process more interesting and motivating. Students are more inclined to actively participate in this interactive learning method. TCLEBL also effectively enhances the students’ clinical thinking and research abilities. By using typical cases as learning materials and requiring extensive literature reviews, TCLEBL cultivates students’ clinical thinking and problem-solving skills, and improves their evidence-based learning methods and clinical/research abilities. This is a combination of the advantages of CBL [24, 25] and EBL [26]. Lastly, TCLEBL combines traditional teacher-led instruction, in which teachers provide guidance to help students grasp key knowledge points, and research methods, consolidating learning outcomes [27].

Additionally, according to the post-course feedback questionnaires, teachers were generally satisfied with TCLEBL. There were several reasons for teacher satisfaction with the TCLEBL teaching method. Firstly, teachers observed that students were actively engaged in learning under TCLEBL and demonstrated high levels of participation and positivity. Secondly, students were able to understand and apply relevant knowledge in case studies, propose reasonable solutions, and demonstrate learning outcomes. Students were able to effectively collaborate, communicate and coordinate within groups to jointly solve case problems. Thirdly, students provided positive evaluations of TCLEBL and offered constructive suggestions and feedback, which teachers found satisfactory and useful to make further improvements.

Furthermore, to more accurately assess the long-term learning outcomes of the postgraduate students, we conducted a theoretical exam two months after the completion of the course and required the submission of a review paper on the disease. In the theoretical exam, students from the TCLEBL group achieved higher scores. This may be attributed to the emphasis of the TCLEBL teaching method on active student learning and participation, enabling them to develop a deeper understanding of the subject matter through group discussions and case analyses in the classroom. This profound understanding helped students better apply and express their acquired knowledge in the theoretical exam, resulting in better grades.

Regarding the review paper on the disease, students from the TCLEBL group achieved higher scores in terms of literature review and citations, arguments and viewpoints, content completeness, scientific rigor, and accuracy. This indicates that the TCLEBL teaching method has significantly contributed to the development of the students’ literature review skills. Through extensive pre-course literature reading and research, students in the TCLEBL group gained a comprehensive understanding of the relevant knowledge related to the disease, enabling them to provide more substantial references and support in their review papers. Furthermore, the TCLEBL teaching method emphasizes the cultivation of students’ arguments and viewpoints. Through group collaboration and discussions, students are encouraged to think deeply and analyze problems, enabling them to express clearer and more compelling arguments and viewpoints in their review papers. Their papers demonstrate greater completeness, covering various aspects of knowledge, and exhibit scientific rigor and accuracy. Review writing plays a crucial role in clinical research. It is an academic writing form that involves comprehensive review, summary, and evaluation of relevant literature on specific topics or areas. In clinical research, reviews are used to systematically integrate and analyze existing research findings, reveal the current state and progress of knowledge, and propose directions and recommendations for future research. The lack of clinical research training for medical graduate students has been a pain point in medical education in China [9, 28, 29]. The TCLEBL teaching method effectively cultivated the students’ skills in literature review through practices that emphasized literature reading, independent research, group collaboration, and academic writing.

Finally, our study found a significant increase in pre-course preparation time for the TCLEBL group. This increase was primarily due to extensive literature reading and evidence searching required by students before the course. This is consistent with the phenomenon of increased extracurricular workload observed in some other new teaching models [30–33]. However, we also observed a significant decrease in post-course review time and review paper writing time for the TCLEBL group. It is noteworthy that despite these changes, the total time spent by students in both groups did not show a significant difference.

However, we must acknowledge some limitations in our study that need to be considered when interpreting the results. Firstly, there may be selection bias as our samples were from a specific school and discipline. Secondly, research results may be impacted by subjective factors in participants. Students’ academic performance and paper quality could be influenced by individual differences, learning motivations, styles, even though controlling for these factors was attempted in the study design. Additionally, our evaluation employed specific assessment methods of theory exams and literature reviews. These may not fully reflect learning outcomes in other areas such as clinical practical skills.

In summary, TCLEBL is an effective teaching method that has achieved significant improvements over traditional teaching models in multiple areas. By conducting learning and collaboration in small groups, TCLEBL promotes classroom interactivity and improves learning efficiency, knowledge acquisition, independent learning ability, and teamwork skills. In addition, TCLEBL focuses on cultivating students’ clinical thinking and research abilities. Using typical cases and extensive literature reviews, students can better understand and apply medical theoretical knowledge to improve clinical practice ability and research competency.

## Data Availability

The datasets used and/or analysed during the current study are available from the corresponding author on reasonable request.
